# Male Horn Lack of Allometry May be Tied to Food Relocation Behaviour in Lifting Dung Beetles (Coleoptera, Scarabaeidae, Eucraniini)

**DOI:** 10.3390/insects10100359

**Published:** 2019-10-18

**Authors:** Claudia Palestrini, Enrico Barbero, Angela Roggero

**Affiliations:** Department of Life Sciences and Systems Biology, University of Torino, I-10123 Torino, Italy; claudia.palestrini@unito.it (C.P.); enrico.barbero@unito.it (E.B.)

**Keywords:** *Anomiopsoides*, static allometry, symmetry, geometric morphometrics, feeding behaviour, arid environment, Argentina endemism

## Abstract

The small dung beetle tribe Eucraniini includes extremely specialized species that have been defined as “lifters” according to their food relocation behaviour. They are characterized by the presence of well-developed expansions on the head and pronotum, which can be included in the large and varied group of horns, whose presence is usually related to complex reproductive tactics. In this study, two closely related species, *Anomiopsoides cavifrons* and *A. heteroclyta*, were examined employing traditional and geometric morphometrics to test whether the Eucraniini has polymorphic males that might exhibit different reproductive tactics, as in the sister tribe Phanaeini, for which a male trimorphism was demonstrated. If also present in Eucraniini polyphenism could be considered a plesiomorphy common to the two clades. The inter- and intraspecific shape variation and object symmetry of the head and the scaling relationships between body size and traits were evaluated. Marked interspecific and small intraspecific differences in shape variation, high symmetry, and similar isometric growth patterns were shown in both species. The hypothesis of male polymorphism in *Anomiopsoides* was thus rejected. Instead, the results supported the alternative hypothesis that Eucraniini lacks male polymorphism, perhaps due to functional constraints affecting the shape of the structures involved in their peculiar food relocating behaviour.

## 1. Introduction

**Figure 1:**
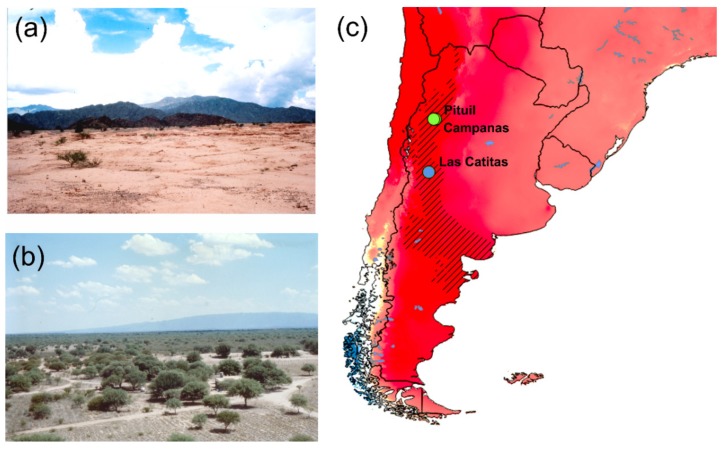
The material collection area: (**a**,**b**) Photos of the landscape features in the La Rojas province, where Pituil and Campanas are located. (**c**) Aridity map, the Monte biogeographical province (NW Argentina) marked by black diagonal lines [4,5,10,11]. The collection localities are marked by blue (Mendoza province, *A. cavifrons*), and green (Las Royas province, *A. heteroclyta*) dots. The Global Aridity Index dataset freely available at CGIAC Consortium for Spatial Information web page (http://csi.cgiar.org/Aridity/) was used to build the map. The aridity degree is shown, where the dark red areas are the aridest.

With only 15 species, all of them endemic to NW Argentina (Figure 1), the tribe Eucraniini (Scarabaeidae, Scarabaeinae) has developed a unique combination of peculiar morphological characteristics and feeding and breeding behaviours [1,2] to survive in a xeric habitat (Figure 1a,b) characterized by extreme aridity and scarce vegetation [2]. The Eucraniini species share a markedly specialized food relocation behaviour, they have adapted to grasp and lift the dry dung pellets of small mammals with their forelegs and carry them into the burrow [2,3,4]. This behaviour, defined as “food-lifting relocation”, is exclusive to Eucraniini among the dung beetles, and characteristically displayed by both sexes [3].

This small coprophagous tribe is currently subdivided into the four genera *Anomiopsoides* Blackwelder, 1944, *Ennearabdus* Lansberge, 1874, *Eucranium* Brullé, 1837 and *Glyphoderus* Westwood, 1837 [3,4,5,6,7], whose systematics were recently reviewed in a thorough survey of their current taxonomic status, nesting behaviour [2], and phylogenetic relationships [8,9].

The laminar, expanded armatures that originate from the clypeal portion of the head are the most conspicuous morphological character in the Eucraniini. Such heavy modifications leading to the development of exaggerated horns [12,13] are usually considered an expression of phenotypic plasticity, as already ascertained for many other Scarabaeinae [14,15,16,17,18].

The shape of the horns is usually quite similar within the Eucraniini, although some differences can be emphasized at the generic level. The males of *Anomiopsoides, Eucranium,* and *Glyphoderus* carry two pairs of elongated horns in the clypeal fronto-lateral part of the head, and the females have similar, but shorter and less developed, horns [3,4,5,7]. In contrast, *Ennearabdus* shows a far less complex head morphology, with only two small horns on the frontal carina of the male while the head of the female is unarmed [6].

The presence of head and pronotum weapons is often linked to complex alternative reproductive tactics in Scarabaeinae males [19,20,21,22]. An exemplar model of such differentiated behaviour is *Onthophagus taurus* (Schreber, 1759), a Scarabaeinae tunneler in which two distinct male morphs have been identified [23]. In this species, it has been demonstrated that each morph has a different strategy in male-male competition, with major males showing aggressive fighting behaviour and minor males showing nonaggressive sneaking behaviour to avoid combat with major males [23]. Notably, both morphs are favoured in different ways in reproductive contests [23].

The cephalic expansions shown by the Eucraniini might be truly regarded as tools involved in male-male competition [22], considering that the tribe is closely related to other Scarabaeinae taxa in which such impressive phenotypic adaptability is not an infrequent occurrence. A recent phylogenetic analysis [24] confirmed that the monophyletic Eucraniini clade constitutes the sister group of the American tribe Phanaeini, which includes more than 150 species at present [25]. Phanaeini are tunnelers that bury dung near, or just below, the dung pad [26,27] and show substantial variation in nesting behaviour [27], as well as a high degree of male polyphenism: males carry a clypeal horn and pronotal prominences that can grow at disproportionate rates compared to overall body growth [28,29,30,31]. The presence of variant male morphs in the same species in response to different factors is an expression of phenotypic plasticity that reflects a varied range of complex situations in the Scarabaeinae [18,32,33,34,35,36]. When allometric relationships between body size and horn length were examined in some Phanaeini species, e.g., *Oxysternon conspicillatum* (Weber, 1801), threshold mechanisms regulating horn expression were detected [37], with size scaling relationships best fitted by a sigmoidal function [38]. In these Phanaeini species, three distinct male phenotypes were found, leading to the identification of alpha, beta, and gamma male morphs, each of which suggested a distinct reproductive strategy that could be defined as a guard, sneak, or mimetic tactics, respectively [37].

The aim of the present research is to test whether the Eucraniini show male polymorphism as seen in the sister tribe Phanaeini [37] and therefore have nonlinear scaling relationships between body size and any of the selected traits (the two clypeal horns and fore tibiae), or else are characterized by isometric body and trait growth [39], which would suggest the action of functional constraints on the developmental patterns of the head and legs. These functional constraints result from their peculiar nesting behaviour. In this framework, the left-right asymmetry of the head [40] will also be evaluated to define any phenotypic variation due to intrinsic and extrinsic factors [41,42] that could affect the object symmetry of the head [43,44]. Two well-characterized, unmistakable and closely related species, *Anomiopsoides cavifrons* (Burmeister, 1861) and *A. heteroclyta* (Blanchard, 1845), have been chosen to examine the shape and size variation by using both traditional and geometric morphometric approaches.

## 2. Materials and Methods

### 2.1. Material

The dataset includes male specimens of *A. heteroclyta* (N_H_ = 16) and *A. cavifrons* (N_C_ = 91), which were collected by EB from north-western Argentina (Las Catitas for *A. cavifrons*, and Pituil and Campanas for *A. heteroclyta*, Figure 1c) during a field expedition in 1989, and housed in the University of Torino, Department of Life Sciences and Systems Biology (MIZT). The collection localities were georeferenced and used to build a map in the GIS environment (QGIS v3.8.2, freely available at https://www.qgis.org/). Also, the Global Aridity Index dataset (freely available at CGIAC-CSI web page, https://cgiarcsi.community/), and the biogeographical regionalisation of Argentina [10,11] (freely available at https://sites.google.com/site/biochartis/) were included in the map.

The specimens were photographed using a Leica DMC4500 digital camera connected to a stereoscopic dissecting scope Leica Z16APO, using the software Leica Application Suite (LAS) to capture and store the images, which were taken carefully avoiding object malpositioning.

A morphological analysis of the head variation was performed applying both traditional and geometric morphometrics approaches. Our analysis focused chiefly on the two pairs of clypeal processes, i.e. the medial (horn1) and lateral (horn2), and the fore tibia.

### 2.2. Traditional Morphometrics Analysis

**Figure 2:**
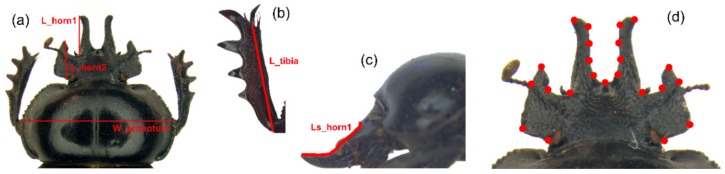
Linear measurements and landmark configuration. (**a**) Maximum pronotal width (W_pronotum), medial and lateral clypeal horn lengths, dorsal view (L_horn1 and L_horn2, respectively). (**b**) Fore tibia length, ventral view (L_tibia). (**c**) Medial clypeal horn length, side view (Ls_horn1). (**d**) Landmark configuration (N_L_ = 23) for the head.

After a careful evaluation, five linear measures (Figure 2a–c) were chosen as reliable estimators of the dimensions of the anatomical traits in the study. The pronotum width is commonly considered a good index of body size in coleopteran taxa [45], while the two head horns and fore tibia were defined by the length linear measurements.

The following measurements (expressed in mm) were taken by the Measurement Module of the software Leica Application Suite (LAS): (1) maximum pronotum width (W_pronotum), as index of body size, (2) medial clypeal horn length, dorsal view (L_horn1), (3) medial clypeal horn length, side view (L_S__horn1), 4) lateral clypeal horn length (L_horn2), 5) right fore tibia length (L_tibia).

Allometric relationships of the length of both clypeal horns, and fore tibia to body size were then tested in males of both species separately [38,45,46] using the software PAST v3.20 [47], and SigmaPlot v10.0 (Systat Software Inc., San Jose, CA, USA, 2007).

The Akaike Information Criterion (aic) was chosen to determine what model best described the allometric relationships (i.e., as the index of goodness of fit) [38]. Additionally, the unimodal or bimodal distribution of data was evaluated using the histogram of the frequency distribution for each measure. All the graphics were made using IBM SPSS Statistics v25 (IBM Corp., Armonk, NY, USA).

For each species, the bivariate analysis was used to evaluate the relationships between each of the four measurements (dependent variables) and the body size (independent variable), and to verify if a common slope could be assigned when analyzing the measured values in pairs [48]. The analysis was done using the software PAST, selecting the RMA regression option.

### 2.3. Geometric Morphometrics Analysis

The geometric morphometrics semilandmark-based approach [49,50,51,52,53,54,55] was applied to describe the head shape variation using tpsDig v2.31 [56] and tpsUtil v1.79 [57] to define the point configuration (Figure 2d). A thorough description of the digitalized landmarks is given in the Supplementary Materials (Figure S1 and Table S1). The criterion for the choice of landmark configuration was to capture at best the overall shape variation of the head.

The head dataset was analyzed by principal component analysis (PCA) using tpsRelw v1.70 [58], examining the species together and separately, to evaluate the overall shape variation, and thus define the variability shown by the two species.

The comparison of the two species was done using tpsRegr v1.45 [59], performing a multivariate test equivalent to a Hotelling generalized T2-Test with 1000 random permutations, and retaining the grids of the two groups to compare the differences in shape variation of the head.

The landmark configuration was then reflected using tpsUtil, to evaluate the symmetry of the head (original vs mirrored configurations, as suggested by Klingenberg [43]), using the software tpsPLS v1.23 [60] with 999 random permutations in the Permutation tests. We focused on the evaluation of shape asymmetry since it can give a more thorough result than the size asymmetry, as suggested by Klingenberg [43] for object symmetry.

## 3. Results

### 3.1. Traditional Morphometric Analysis

**Figure 3:**
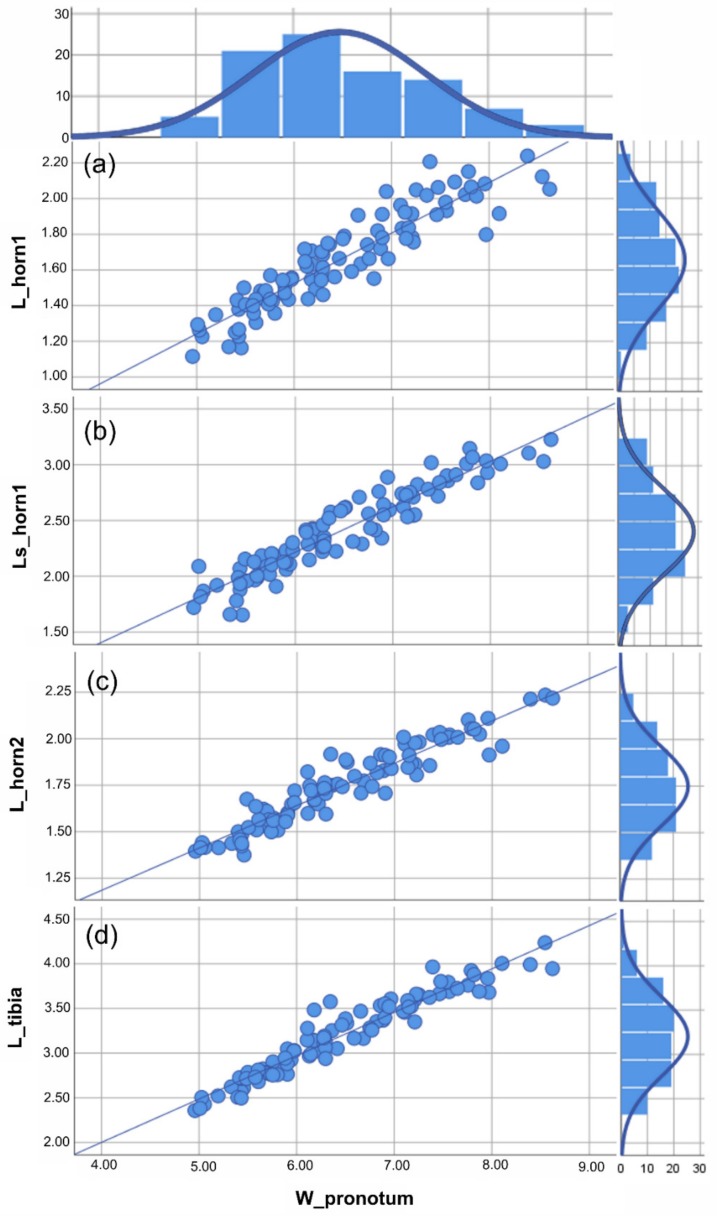
*Anomiopsoides cavifrons* size analysis. All the measures were expressed in mm. For each linear measure plotted against the body size (i.e., W_pronotum), the scatterplot with the best fitting line (defined by the lowest aic value) and histogram of the frequency of individuals are given. (**a**) Scatterplot of the medial clypeal horn length, dorsal view, and body size, the aic value = 5.147 (linear function). (**b**) Scatterplot of the medial clypeal horn length, side view, and body size, the aic value = 5.629 (linear function). (**c**) Scatterplot of lateral clypeal horn length, dorsal view, and body size, the aic value = 4.556 (linear function). (**d**) Scatterplot of the fore tibia length, ventral view, and body size, the aic value = 5.542 (linear function). The descriptive statistics (Table 1) are discussed in the text. See Supplementary Materials Table S2 for the statistical values of the graphics.

**Figure 4:**
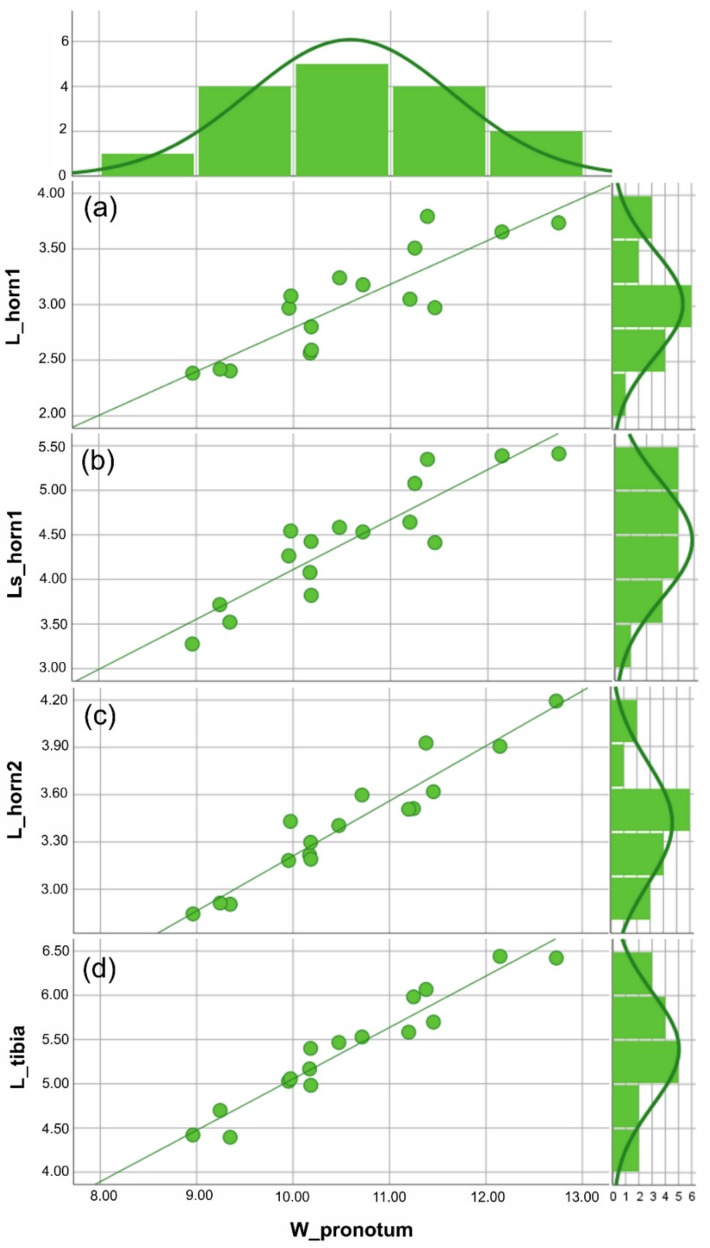
*Anomiopsoides heteroclyta* size analysis. All the measures were expressed in mm. For each linear measure plotted against the body size (i.e., W_pronotum), the scatterplot with the best fitting line (defined by the lowest aic value) and a histogram showing the frequency of individuals are given. (**a**) Scatterplot of the medial clypeal horn, dorsal view, and body size, the aic value = 5.796 (linear function). (**b**) Scatterplot of the medial clypeal horn, side view, and body size, the aic value = 6.158 (linear function). (**c**) Scatterplot of lateral clypeal horn, dorsal view, and body size, the aic value = 5.115 (linear function). (**d**) Scatterplot of the fore tibia length, ventral view, and body size, the aic value = 5.344 (linear function). The descriptive statistics (Table 1) are discussed in the text. See Supplementary Materials Table S2 for the statistical values of the graphics.

Based on the traits examined here (i.e., the two clypeal horns and fore tibiae), different male morphs could not be identified in either *A. cavifrons* or *A. heteroclyta*. For the two species, analyses of the scaling relationship between each of the traits and body size always gave a better fit for the linear function than for Hill’s sigmoid function, according to the AIC values (Figure 3 and Figure 4, Supplementary Materials Table S2).

The descriptive statistics of the linear measures (Table 1) showed that although *A. heteroclyta* was larger than *A. cavifrons*, both species showed a similar pattern for all measurements. Furthermore, *A. heteroclyta* had a slightly higher variance in body size (W_pronotum) than in the other measures.

The comparison of the regressions (RMA) suggested a common pattern for both horn and fore tibia measurements plotted against body size, with *p*_(same slope)_ < 0.001, when *A. cavifrons* and *A. heteroclyta* were examined separately.

### 3.2. Geometric Morphometric Analysis

**Figure 5:**
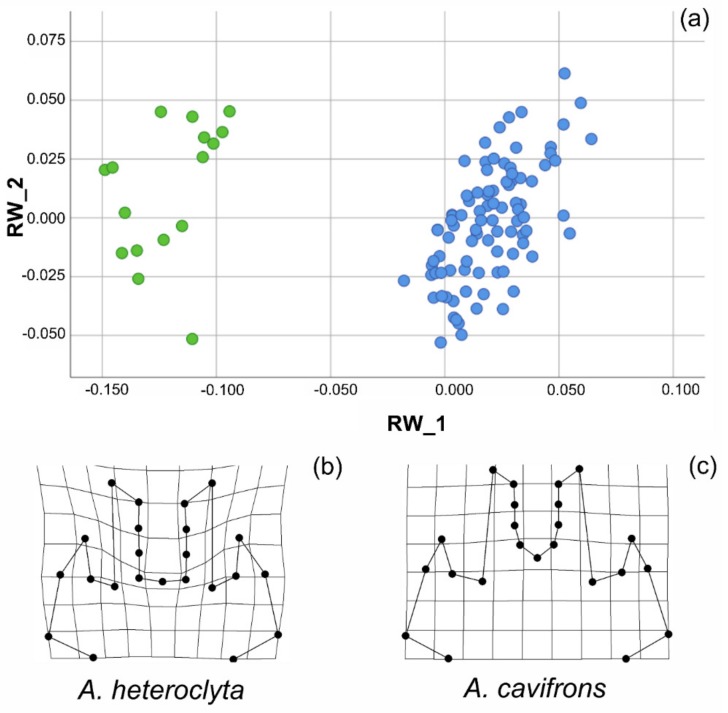
Shape analysis of *Anomiopsoides* male head. (**a**) Scatterplot of the RWs 1 and 2, which accounted together for about 73% of the overall shape variation of the head. (**b**) Deformation grid of the *A. heteroclyta* group from the tpsRegr analysis. (**c**) Deformation grid of the *A. cavifrons* group from the tpsRegr analysis.

In the analysis of the head, the landmark configuration (N_H_ = 23) was analysed using PCA, with the first three out of 42 relative warps (i.e., the principal components hereinafter referred to as RWs) explaining almost 73% of the overall variation in shape. Only the first three RWs explained > 5.0% of the variation in shape. The two species were characterized by extremely differentiated heads, as shown in the scatterplot of RWs 1 and 2 (Figure 5a). The deformation grids (Supplementary Materials Figure S2) demonstrated marked shape differences in axes extremities. When the two species were analysed separately, it was instead demonstrated that the overall variation in head shape was very small, with each species showing homogeneous male weaponry. For example, the cumulative percentage of the overall shape variation explained by relative warps 1 and 2 was only 48.85% for *A. cavifrons*, extending the morphospace in the scatterplot (Supplementary Materials Figure S2) from -0.063 to 0.162 for RW_1 (range = 0.225, variance = 0.004) and from−0.055 to 0.052 for RW_2 (range = 0.107, variance = 0.001).

The marked overall differentiation in head features between the two species was confirmed by the results of the multivariate test of significance (Wilks’ Lambda: F = 90.596, df = 42, 64.0 *p* < 0.01, Generalized Goodall F-test: F = 113.978, df = 42, 441, *p* = 0.000, and Permutation tests: percent of Goodall F values ≥ observed = 0.10%), as shown by the deformation grids of the two groups (Figure 5b,c).

When the symmetry of the head was analysed using the partial least-square analysis in tpsPLS (original vs. reflected configurations), a significant result was found (Table 2) for the correlation of the two sides of the head for both species. Similar results showing only a subtle asymmetry [40] were obtained. The cross-set analysis gave, as usual, the most significant value of covariation for only one of the calculated dimensions (D1, Table 2), whereas the other dimensions had negligible percent values of covariance and thus could be discarded. It was noteworthy that, although D1 accounted for the majority of the covariance, all the dimensions showed a high correlation (expressed by the r value) between the two shape configuration vectors, with individuals always disposed around the midline in the plots. The r value for all the dimensions was >0.99 for *A. heteroclyta* and >0.91 for *A. cavifrons*, meaning that the strength of the linear relationship between the shapes of the head and head-reverted was very high in the two species. The present results, therefore, demonstrated a high degree of symmetry in the structure, as also confirmed by the statistical results for shape projections (Table 2).

## 4. Discussion

According to the results of the analyses, the presence of two pairs of expanded cephalic horns in *Anomiopsoides* cannot be considered an example of polyphenic development related to male-male reproductive competition, as seen in the sister tribe Phanaeini [37], since different male morphs were not detected in the tribe Eucraniini. The hypothesis that male polymorphism should be considered a plesiomorphic character shared by these two sister clades must, therefore, be rejected. Noteworthy, within the Eucraniini species, *Ennearabdus lobocephalus* (Harold, 1868) shows far less developed head projections, and is considered the most primitive species within the tribe [6,8], suggesting thus that the extreme development of clypeal horns showed by the other species of this tribe is a derivative condition. An alternative hypothesis that could be supported by the present finding is that morphological integration of traits depends on functional constraints. The proposed hypothesis suggested a much different scenario for the development of such shaped horns in Eucraniini, in which distinct male morphs were not identified and isometric growth related to body size for the horns and fore tibiae was detected, suggesting a pattern of proportional scaling relationships between those structures. The present results may be explained by considering that both horns and fore tibiae cooperate in the peculiar food-lifting relocation behaviour of these dung beetles [27].

The congruent linear scaling relationships highlighted for all the chosen traits in relation to body size (Figure 3 and Figure 4) could be a common developmental pattern in Eucraniini, with larger individuals having longer horns and fore tibiae and possibly carrying larger pellets. Smaller individuals sometimes cut in pieces the dung to move it easier [2]. Comparing the two species, overall dimensional differences were detected (Table 1), but both species expressed similar patterns in the expression of static allometry in the scaling relationships between body size and traits (Figure 3 and Figure 4). In Eucraniini the two structural traits involved in food-lifting relocation are characterized by an isometric growth [38,46,61], and these results can suggest a functional link between the clypeus and fore tibiae, which may be regarded as a developmental constraint [62].

The morphological integration of traits [63,64] is a relatively common occurrence in holometabolous insects, in which, due to the determinate mechanism of growth, the highest phenotypic correlation can be found [65]. For Eucraniini, a functional interaction between head and foreleg development can be proposed, with related traits being affected in the same way [65] and static allometry often being strongly constrained [66]. As a rule, functional constraints that affect the potential growth of traits can reduce the expression of allometry. Thus, functionally related traits cannot vary freely [63,67].

It is not always easy to determine which factors regulate the relationships between traits, although nutritional regulation is surely involved in insect development [61,68,69,70]. Usually, changes in nutrition can greatly affect adult body size and traits across and within species, with many examples being found in the Scarabaeinae [61,71]. In the Eucraniini, the scarcity and physical characteristics of resources could have reduced the range of nutritional effects on phenotypic size variability. These species neither modify the collected pellets in any way nor exhibit soil lining behaviour, i.e., the dung is not manipulated at all within the nest [27]. Maternal effects related to food relocation might not be involved in the modulation of the expressed offspring phenotypes in this tribe [19]. Thus, the development of exaggerated cephalic horns might not be affected by growth regulation depending on the allocation of diversified food resources [19]. An equally expanded cephalic armature (hyperallometry) is present in all *Anomiopsoides* males, thus, different male morphs cannot be detected in the tribe Eucraniini. Therefore, the appearance of any alternative reproductive tactics expressed by di- or even trimorphic males [23,37,72] cannot be accounted for here. Although the biology of the Eucraniini is not known in detail, some kind of sexual cooperation has been observed during the provisioning of the underground chamber, which involves occasional external patrolling near the entrance of the tunnel, an activity that is performed indifferently by either individual of the couple [2] to watch over the food resources and avoid depredation. These observations [2,73,74] supported the hypothesis that males may not have developed any true “guard tactics” to protect the entrance of the tunnel with the breeding female inside the nest, as observed in other dung beetles that exhibit male polyphenism [34,38].

Among the Scarabaeinae, the Eucraniini are characterized by a very peculiar, distinctive morphology, with all the body parts being highly modified, and the species within this tribe are all well characterized, distinct and easily identifiable. The head is nevertheless the most diversified morphological trait, in which the differential pattern of variability is expressed both within and among the species. The shape analysis showed higher variation at the interspecific level compared to the intraspecific level for the male head, which is thus relatively uniform within each species but clearly differentiated between the species (Figure 5a). While the overall variation in shape defined distinct patterns at the species level, differently shaped morphs could not be detected within each species, as they showed continuous rather than discrete variability patterns, which was also evidenced by the deformation grids (Supplementary Materials Figure S2) when comparing the overall shape variation at the species level.

The majority of the shape variation at the interspecific level was related to modifications of the anterior part of the head, depending not only on the medial horns, which are extremely different in the two species, but also on the central area within the medial horns (corresponding to the area between points 11 and 13, see Supplementary Materials Figure S1), which is modified differently in the two species (Figure 5b,c). The lateral horns displayed a less marked variation (Figure 5a, Supplementary Materials Figure S2). The development of horns can ostensibly affect other parts of the head, although the underlying processes of these correlated modifications have not yet been fully characterized [64]. Additionally, within each species, the majority of shape variation was noted in the same, very circumscribed, portion of the head (Supplementary Materials Figure S2). The medial horns were also slightly different within each species, but the amount of overall shape variation was lower and related, for example, to small variations in the apex or in the expanded area at the base of the medial horns. The particular feeding behaviour of this tribe influenced not only the size but ostensibly also the shape variation of the head, and it was involved together with the forelegs in lifting and transporting the dung pellets.

The results of the analyses that tested the presence and degree of asymmetry in both *Anomiopsoides* species confirmed the strength of the functional constraints defining the pattern of shape variation of the head. This structure showed an elevated symmetry in Eucraniini, as expected in structures that are characterized by strong functionality and hence subject to functional constraints [75]. The maintenance of the developmental stability of the clypeal projections is important in food-lifting relocation involving the head horns and fore tibiae [27].

The Eucraniini head manifested a pattern of fluctuating asymmetry [43] with developmental errors producing an asymmetry that was normally distributed around a mean of zero [40]. No statistically significant directional asymmetry was detected, unlike that reported for a wide range of organisms, including insects [43].

Fluctuating asymmetry can be used to infer the developmental origin of integration within morphological structures through a geometric morphometric approach [43]. The present results, which show a low degree of asymmetry in the head, support that the two medial horns are functionally correlated and that large differences between the left and right averages might be disadvantageous for this structure. It could, therefore, be suggested that some kind of constraint prevents the expression of greater asymmetry in the head during development. The Eucraniini do indeed exhibit strong developmental stability against adverse phenotypic variations, which could affect food relocation performance, reducing the effectiveness of dung pellet removal.

## 5. Conclusions

To summarize, the hypothesis that the Eucraniini might show male polymorphism was rejected since isometric relationships were detected between body size and each of the selected traits. The head showed well-differentiated patterns of shape variation for the two *Anomiopsoides* species and a low degree of asymmetry in both species. Thus, no different shape morphs were identified. In the Eucraniini, low shape variability of the structures of the clypeus was shown, which can favour their functional performance. In this framework, we can, therefore, assume that male clypeal morphology and the peculiar food relocating behaviour of the Eucraniini evolved in concert. Thus, we support the alternative hypothesis that the exaggerated and even growth of clypeal horns is linked to—and constrained by—functionality in the Eucraniini. In this tribe, also the females carry evident clypeal horns, although not so greatly developed as in males. Since we focused on male polyphenism, the horns of females were not examined here. However, a thorough examination of the size and shape variation of female horns could contribute to elucidate the developmental mechanism of the head structures within the Eucraniini.

## Figures and Tables

**Table 1:** Descriptive statistics of linear measurements in the males of both *Anomiopsoides* species.

		Range	Min	Max	Mean	Std Error	Std Dev	Variance
***cavifrons***	**W_pronotum**	3.664	4.958	8.622	6.472	0.092	0.886	0.786
	**L_horn 1**	1.124	1.115	2.239	1.658	0.028	0.272	0.074
	**Ls_horn 1**	1.573	1.653	3.226	2.409	0.040	0.383	0.147
	**L_horn 2**	0.860	1.375	2.235	1.748	0.022	0.213	0.045
	**L_tibia**	1.880	2.357	4.237	3.201	0.047	0.448	0.201
***heteroclyta***	**W_pronotum**	3.769	8.961	12.730	10.585	0.262	1.049	1.101
	**L_horn 1**	1.409	2.383	3.792	3.020	0.119	0.477	0.227
	**Ls_horn 1**	2.135	3.276	5.411	4.440	0.163	0.654	0.427
	**L_horn 2**	1.350	2.844	4.194	3.415	0.095	0.382	0.146
	**L_tibia**	2.048	4.394	6.442	5.396	0158	0.633	0.401

**Table 2:** Results of the PLS analysis and subsequent Permutation Tests (nReps = 999) for D1 in both *Anomiopsoides* species (shape1 = original, shape2 = reflected).

Name of the Specie	Cross Set Analysis	r	Statistics for Shape1 Projections			Statistics for Shape2 Projections			Permutation Tests
			Min	Max	Std Dev	Min	Max	Std Dev	% of Correlations ≥ Observed
***cavifrons***	77.363	0.998	−0.077	0.053	0.028	−0.077	0.052	0.027	0.10
***heteroclyta***	69.536	0.999	−0.047	0.066	0.034	−0.047	0.066	0.034	0.10

## Supplementary Materials

The following are available online at https://www.mdpi.com/2075-4450/10/10/359/s1, Figure S1: Configuration of the points of the head, Table S1: Description of the landmarks digitized on the head of the *Anomiopsoides* males, Table S2: Stats values of the graphics in Figure 3 and Figure 4, Figure S2: Scatterplot of the head, with the deformation grids of the two species.
