# Supplementary material for: Male Horn Lack of Allometry May be Tied to Food Relocation Behaviour in Lifting Dung Beetles (Coleoptera, Scarabaeidae, Eucraniini)
Source: Insects. 2019 Oct 18;10(10):359. doi: 10.3390/insects10100359 (PMC6835258; doi:10.3390/insects10100359)
Supplement: Supplementary file 1 [file insects-10-00359-s001.pdf]

Supplemental material – Figure S1 and Table S1

Description of the landmarks digitized on the head of the *Anomiopsoides* males

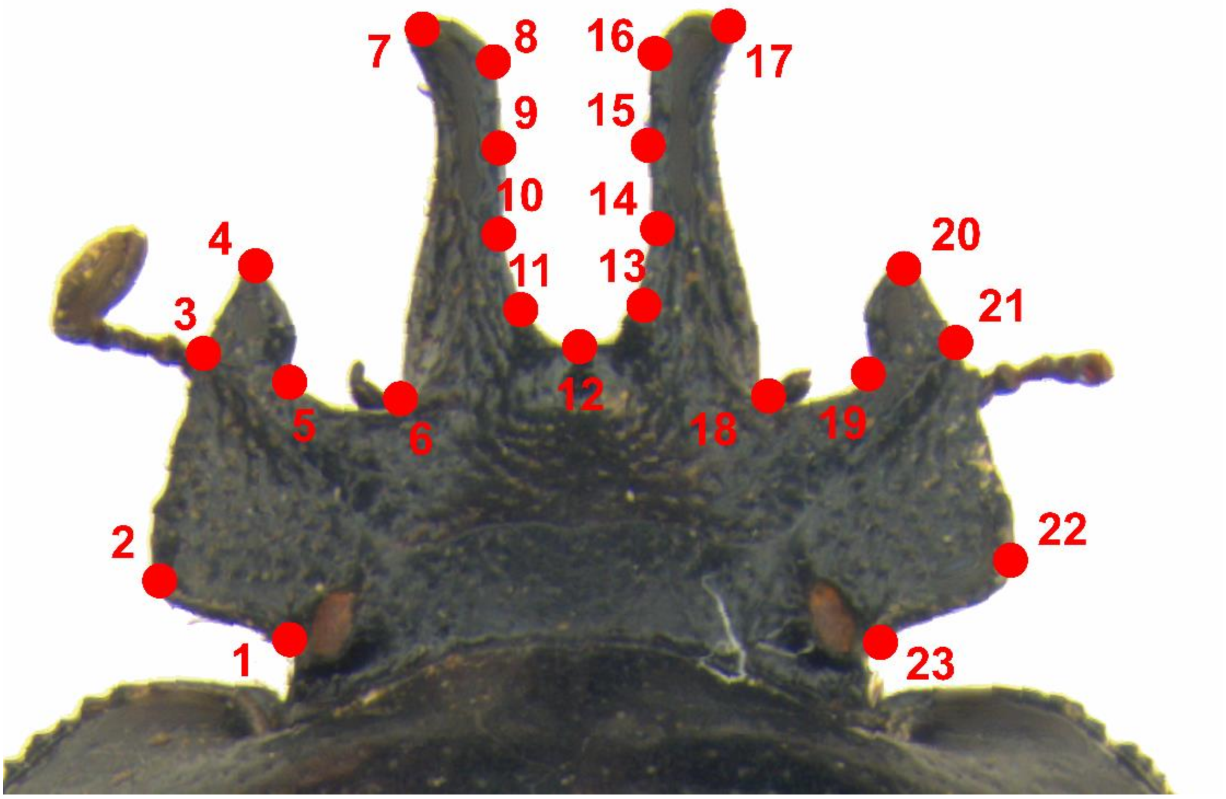

No	Landmark definition
**1**	Base of the head, near the eye angle, left
**2**	Left gena, base
**3**	Genal carina, left
**4**	Apex of the horn 2, left
**5**	Inner base of the horn 2, left
**6**	Outer base of the horn 1, left
**7**	Apex of the horn 1, left
**8-11**	Inner margin of horn 1, left (semilandmarks)
**12**	Anterior midpoint of the head
**13-16**	Inner margin of horn 1, right (semilandmarks)
**17**	Apex of the horn 1, right
**18**	Outer base of the horn 1, right
**19**	Inner base of the horn 2, right
**20**	Apex of the horn 2, right
**21**	Genal carina, right
**22**	Right gena, base
**23**	Base of the head, near the eye angle, right

Supplemental material – Table S2

Statistics of the graphics in Figs 3 and 4. The goodness of fit was calculated using the software PAST, here the AIC value of linear function and Hill'sigmoid function are given. The power function slope was calculated on the scatterplots by SPSS; the 95% confidence intervals were obtained by SPSS (one-sample T-test, with P<0.000 for all the analyses). The 95% confidence interval for the pronotum width were 6.288/6.657 for *A. cavifrons*, and 10.026/11.144 for *A. heteroclyta*.

		goodness of fit		power function slope		95% confidence interval	
		AIC_L	AIC_H	linear function	R^2	lower	upper
cavifrons	**L_horn1**	5.147	9.372	y=0.17+0.28*x	0.848	1.601	1,714
	**L_horn2**	4.556	8.869	y=0.28+0.23*x	0.897	1.704	1.793
	**Ls_horn1**	5.629	9.881	y=0.22+0.41*x	0.887	2.330	2.498
	**L_tibia**	5.542	9.639	y=0.06+0.49*x	0.922	3.108	3.294
heteroclyta	**L_horn1**	5.796	12.477	y=1.13+0.39*x	0.744	2.766	3.275
	**L_horn2**	5.115	11.827	y=0.28+0.35*x	0.913	3.212	3.620
	**Ls_horn1**	6.158	12.734	y=1.49+0.56*x	0.808	2.766	3.275
	**L_tibia**	5.344	12.002	y=0.76+0.58*x	0.930	5.059	5.733

Supplemental material – Figure S2

On the scatterplot of RWs 1&2 the deformation grids corresponding to the extreme values of the x-y axes are showed, the position being marked by the red stars. The consensus confuguration is below, its position at crossing point of the axes (coordinates 0.000, 0.000) marked by a black star. For each species, the individual grids used to show the overall shape variation (see below) are marked by a star, and numbered.

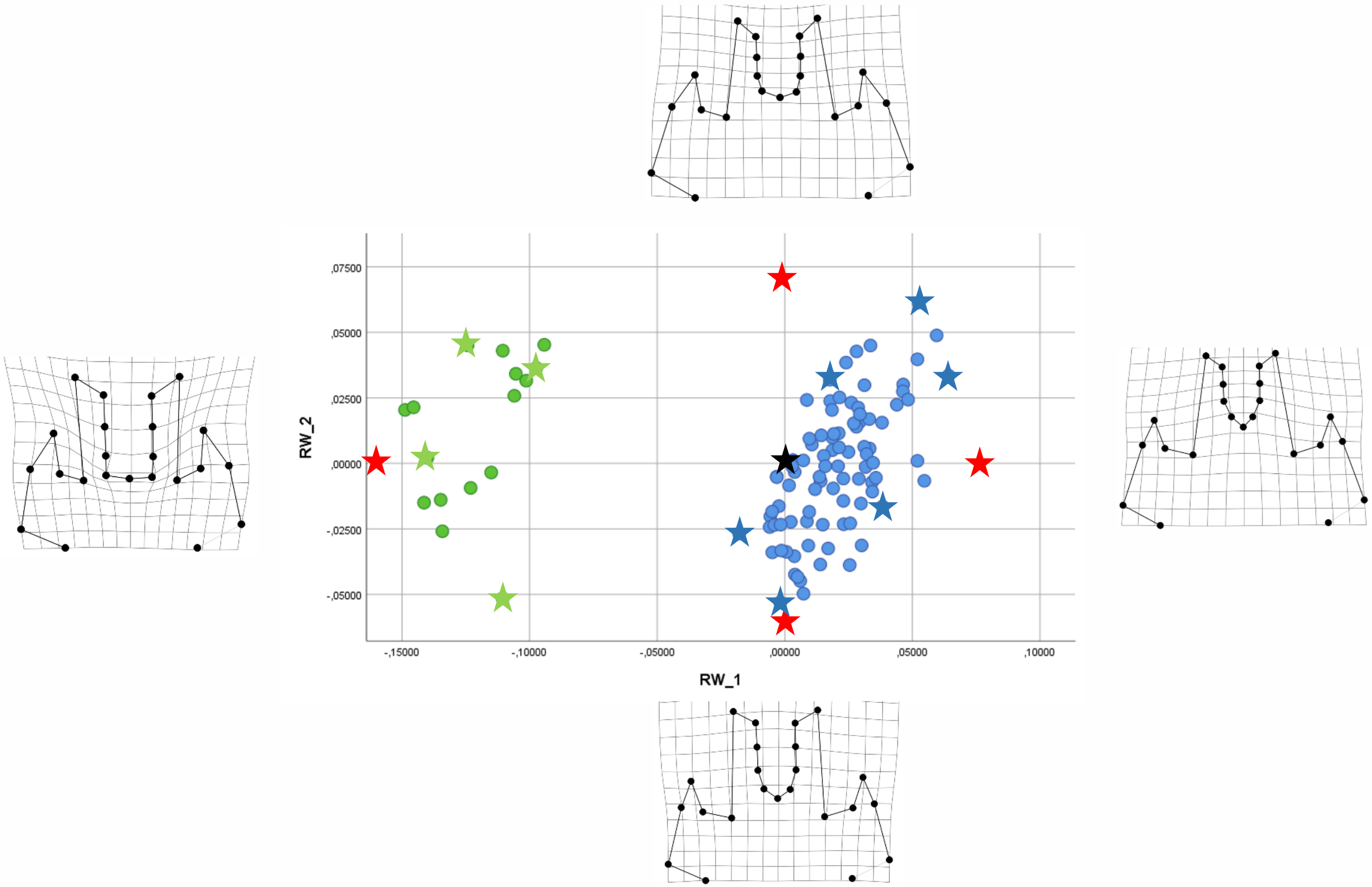

Consensus configuration of the RWs 1 and 2

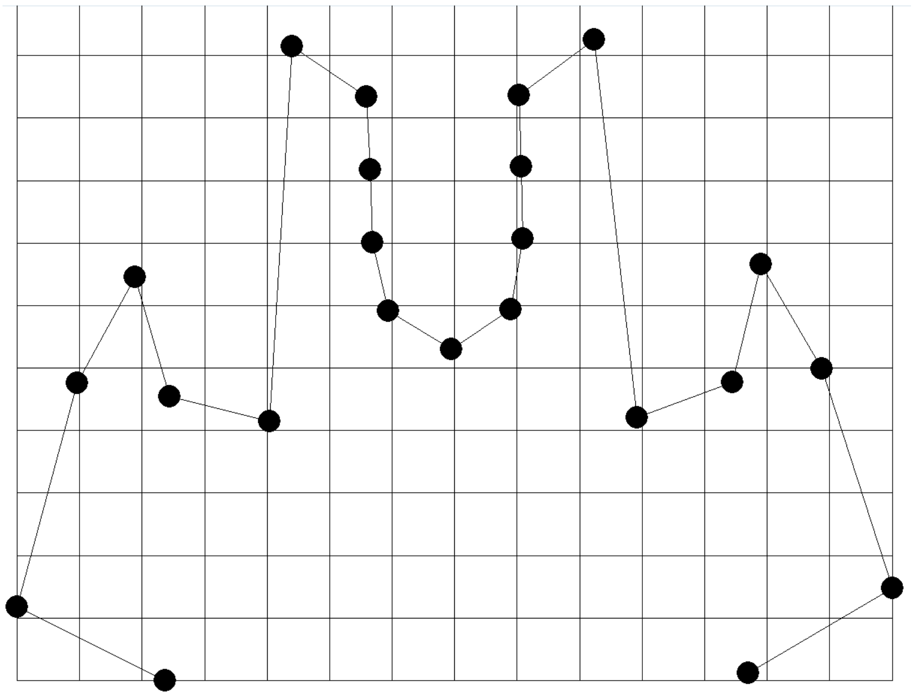

***Anomiopsoides heteroclyta* shape variability**

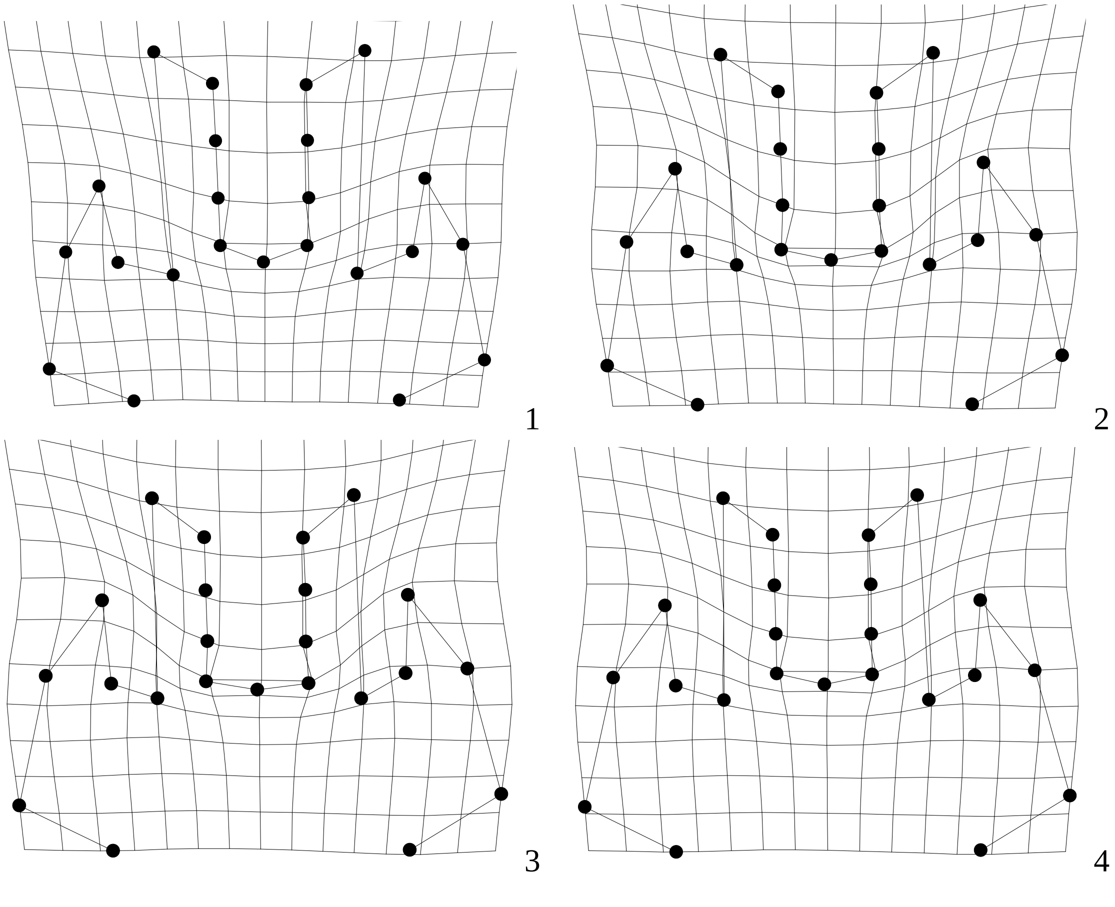

In *A. heteroclyta* the shape variation showed by the plot of RW 1 & 2 is clearly related to the horn 1 variation, while the horn 2, the genae and the basal part of the head do not vary, although the horn 2 could be slightly inward turned. The analysis showed that the head is markedly symmetrical.

Anomiopsoides cavifrons **shape variability**

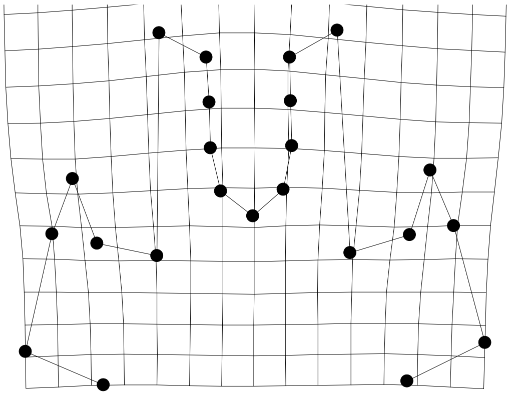

5

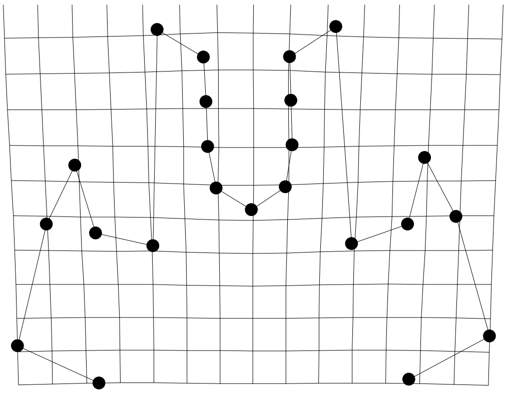

6

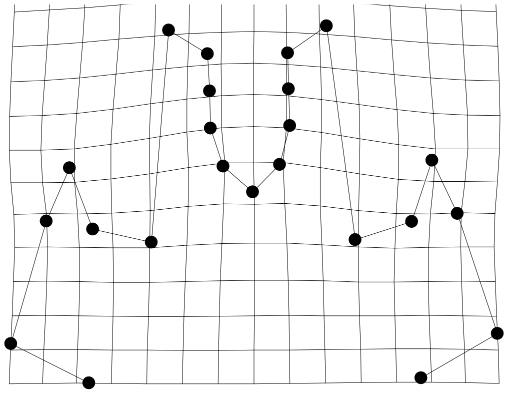

7

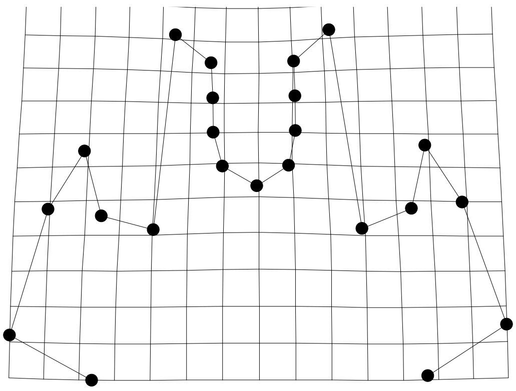

8

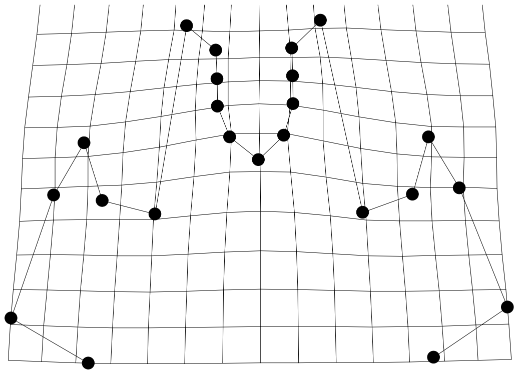

9

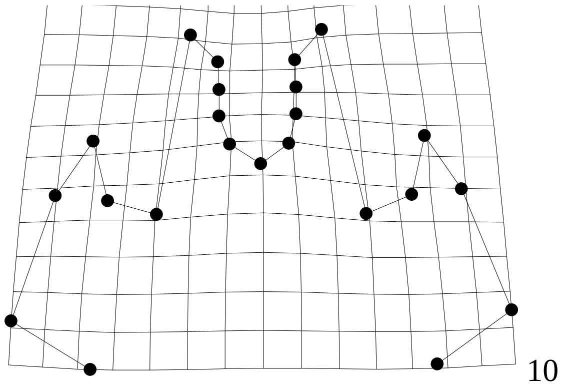

10

Also in *A. cavifrons* analogous results to those already showed by *A. heteroclyta* were obtained. While the horn 2, the genae and the basal part of the head do not much vary, the shape variation showed by the plot of RW 1 & 2 mainly concerned the horn 1, which show a higher variation than in the former species, although the head remain markedly symmetrical also in this species. A high degree of variation is also displayed by the anterior part ogf the head between the two medial horns (i.e., horn 1) which can be very differently shaped.
